# Supplementary material for: MetaRibo-Seq measures translation in microbiomes
Source: Nat Commun. 2020 Jun 29;11:3268. doi: 10.1038/s41467-020-17081-z (PMC7324362; doi:10.1038/s41467-020-17081-z)
Supplement: Supplementary file 10 — Supplementary Data 7 [file 41467_2020_17081_MOESM10_ESM.zip › File2/Confidence_High_Taxonomy/91503_out.krona.html]

Javascript must be enabled to view this page.

members
magnitude
magnitudeUnassigned
count
unassigned
taxon
rank

91503\_out

2

2
superkingdom
2

1239
phylum
2

2
class
91061

order
2
186826

1300
2
family

1301
genus
2

1304
1

SRS020856\_contig\_number\_contig-100\_207.102240.102240
species

1

SRS148152\_contig\_number\_contig-100\_2265.26807
species
28037
